# O020: A new method of bacteriophage-based disinfection in healthcare settings

**DOI:** 10.1186/2047-2994-2-S1-O20

**Published:** 2013-06-20

**Authors:** EB Brusina, OM Drozdova, AG Kutikhin

**Affiliations:** 1 Department of Epidemiology, Kemerovo State Medical Academy, Kemerovo, Russian Federation

## Introduction

One of the most significant features of healthcare-associated infections (HAIs) is the high frequency of multidrug-resistant bacterial strains (MDRBSs). Even modern chemical antimicrobials (CAMs) are not efficient enough; a totally different way of prevention of HAIs caused by MDRBSs is necessary.

## Objectives

To develop a monobacteriophage (MBP)-based way of prevention of HAIs caused by MDRBSs.

## Methods

MBPs against *Pseudomonas aeruginosa* (PA), methicillin-resistant *Staphylococcus aureus* (MRSA), *Salmonella spp.*, *Shigella spp.*, and *Klebsiella spp.* were sprayed on various surfaces in different healthcare settings (HCSs) in the case of spread of HAIs caused by these agents. We compared efficiency of MBPs on different surfaces and in different conditions of phage circulation. Finally, we assessed the influence of the MBPs on incidence and mortality of HAIs caused by MDRBSs. The efficiency of the MBPs was evaluated in the terms of absence of the target bacteria in the environment, that, in turn, was assessed by classical bacteriological methods.

## Results

Application of MBPs sprayed on surfaces in hospital environment is significantly more efficient method of elimination of MDRBSs compared to their usage by any other way. MBPs possessed greater efficiency on glass, metal, and plastic surfaces compared to textile and paper. Duration of MBP circulation was determined by time frame, MBP strain, and lytic activity of the MBP, but not by dose of MBP on the surface. The greatest effect was revealed against PA; even the single usage of MBP provided total elimination of PA from the hospital environment. The application of MBPs led to 15-fold decrease of incidence of *Salmonella*-caused infections, 4-fold reduction of incidence of *Shigella*-caused infections, and 2-fold decrease of incidence of *Klebsiella*- and MRSA-caused infections. Finally, CAMs did not influence the efficiency of MBPs, and no side effects were registered.

## Conclusion

The MBP-based way of prevention of HAIs caused by MDRBSs has certain advantages over CAMs. It may be used without limitations in different HCSs, particularly in intensive care units, it allows the efficient elimination of MDRBSs from the hospital environment in short terms, it provides termination of the outbreaks caused by MDRBSs, reduces the incidence and mortality from HAIs, and it is much cheaper compared to CAMs.

## Disclosure of interest

None declared.

